# Supplementary material for: A Tunable Nanoplatform of Nanogold Functionalised with Angiogenin Peptides for Anti-Angiogenic Therapy of Brain Tumours
Source: Cancers (Basel). 2019 Sep 6;11(9):1322. doi: 10.3390/cancers11091322 (PMC6770958; doi:10.3390/cancers11091322)
Supplement: Supplementary file 1 [file cancers-11-01322-s001.pdf]

Supplementary Materials

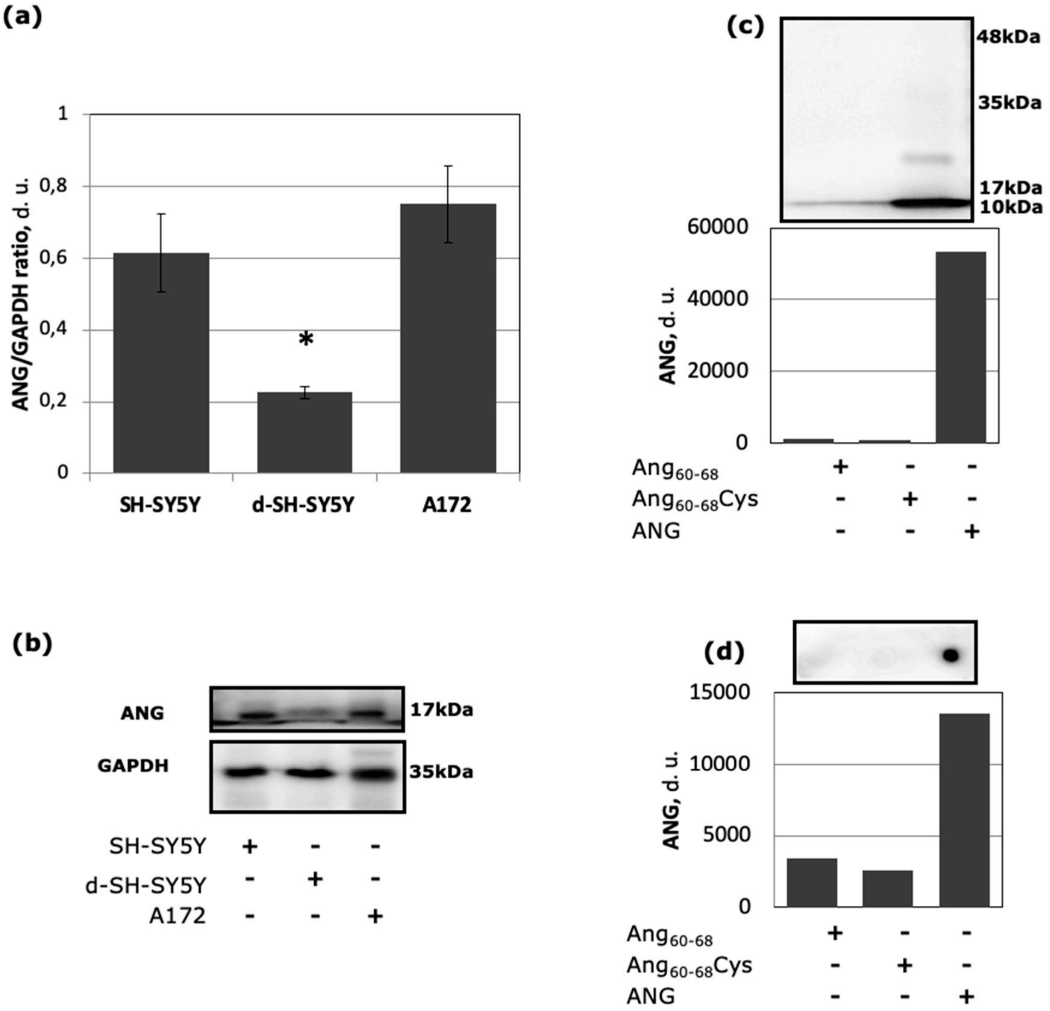

Figure S1. Densitometric analyses (**a**) and representative Western blot (**b**) of SH-SY5Y, differentiated SH-SY5Y and A172 cell lysates. Representative Western (**c**) and dot (**d**) blotting of $Ang_{60–68}$, $Ang_{60–68}$Cys and ANG. Nitrocellulose membranes were incubated with anti-angiogenin antibody. Each dot for Dot Blot analysis was loaded with 2 µl of protein or peptide samples with concentration 0.2 mg/mL. The bars represent means ± SD of three independent experiments performed in triplicate (S.D. = standard deviation). Statistically significant differences, determined by one-way ANOVA are indicated: * $p \leq 0.05$ versus both, undifferentiated SH-SY5Y and A172 cells.
